# The Golgi associated ERI3 is a *Flavivirus* host factor

**DOI:** 10.1038/srep34379

**Published:** 2016-09-29

**Authors:** Alex Michael Ward, Meredith E. K. Calvert, Leah R. Read, Seokyoung Kang, Brandt E. Levitt, George Dimopoulos, Shelton S. Bradrick, Jayantha Gunaratne, Mariano A. Garcia-Blanco

**Affiliations:** 1 Program in Emerging Infectious Diseases, Duke-NUS Graduate Medical School, Singapore; 2 Bioimaging & Biocomputing Facility, Temasek Lifesciences Laboratory, Singapore; 3 W. Harry Feinstone Department of Molecular Microbiology and Immunology, Bloomberg School of Public Health, Johns Hopkins University, Baltimore, Maryland, USA; 4 Center for RNA Biology and Department of Molecular Genetics and Microbiology, Duke University Medical Center, Durham, North Carolina, USA; 5 Department of Biochemistry and Molecular Biology, The University of Texas Medical Branch, Galveston, Texas, USA; 6 Translational Biomedical Proteomics Laboratory, Institute of Molecular and Cell Biology, Singapore; 7 Yong Loo Lin School of Medicine, National University of Singapore, Singapore; 8 Department of Medicine, Duke University Medical Center, Durham, North Carolina, USA

## Abstract

Dengue virus (DENV) is a mosquito-borne *Flavivirus* classified into four serotypes (DENV-1-4) that causes Dengue fever (DF), Dengue hemorrhagic Fever (DHF) or Dengue shock syndrome (DSS). An estimated 390 million people are at risk for infection with DENV and there are no effective vaccines or therapeutics. We utilized RNA chromatography coupled with quantitative mass spectrometry (qMS) to identify host RNA binding proteins (RBPs) that interact with DENV-2 RNA. We identified ERI3 (also PRNPIP and PINT1), a putative 3′–5′ RNA exonuclease, which preferentially associates with DENV-2 genomic RNA via interactions with dumbbell structures in the 3′ UTR. ERI3 is required for accumulation of DENV-2 genomic RNA and production of infectious particles. Furthermore, the mosquito homologue of ERI3 is required for DENV-2 replication in adult *Aedes aegypti* mosquitos implying that the requirement for ERI3 is conserved in both DENV hosts. In human cells ERI3 localizes to the Golgi in uninfected cells, but relocalizes near sites of DENV-2 replication in infected cells. ERI3 is not required for maintaining DENV-2 RNA stability or translation of the viral polyprotein, but is required for viral RNA synthesis. Our results define a specific role for ERI3 and highlight the importance of Golgi proteins in DENV-2 replication.

Dengue viruses (DENV), which are four mosquito-borne *Flaviviruses* distributed throughout the tropics, typically cause classical dengue fever (DF), but can also cause dengue hemorrhagic fever (DHF) or dengue shock syndrome (DSS)^1^,^2^. Other *Flaviviruses* include the Yellow fever virus (YFV), West Nile virus (WNV), the Japanese encephalitis virus (JEV), and Zika virus (ZIKV)^3^.

The DENV genome is a capped, non-polyadenylated positive-strand RNA that encodes three structural proteins (C, prM and E) and seven nonstructural proteins (NS1, 2A, 2B, 3, 4A, 4B and 5)^3^. DENV proteins are translated as a single polyprotein that is cleaved by cellular and virus-encoded proteases to give rise to individual DENV proteins. Viral RNA replication is accomplished by the multifunctional proteins NS3 and NS5, which copy viral RNA into negative strand templates for subsequent rounds of positive strand RNA amplification. Nascent transcripts are utilized as templates for translation of viral proteins, packaged into newly assembled virions or degraded by cellular enzymes to generate the 3′ UTR-derived subgenomic flaviviral RNA (sfRNA)^3^,^4^.

Viral replication occurs in close association with intracellular membranes in part to conceal dsRNA replication intermediates, which can be detected by pattern recognition receptors (PRRs) and induce a strong innate immune response to infection^5^,^6^. While several studies suggest that these membranes are derived from the endoplasmic reticulum, the dynamics and precise composition of membranes that support DENV replication are not known^6^,^7^. Indeed, several studies using the Kunjin strain of WNV demonstrate that Golgi-derived membranes are recruited to replication sites^8^,^9^. Host cell proteins that establish and maintain interactions between intracellular membranes, viral RNA and sites of viral replication are essential for viral replication and are attractive targets for therapeutic intervention.

The DENV untranslated regions (UTRs) contain secondary structures that are required for replication and act as sites of assembly for host cell proteins^10^,^11^. We utilized RNA chromatography coupled with quantitative mass spectrometry to identify proteins that bind to the DENV-2 UTRs^10^,^12^. We identified ERI3 (also PRNPIP or PINT1) in an RNA chromatography-based proteomics screen host proteins that interact with viral RNAs. ERI3 was previously identified in a yeast two-hybrid screen for proteins that interact with prion protein and is closely related to ERI1 (also 3′hExo), a 3′–5′ RNA exonuclease that regulates 3′ end formation and cell cycle-dependent turnover of histone mRNAs^13^,^14^,^15^,^16^. In addition, ERI3 was identified in an RNAi screen for proteins required for early events in HIV-1 replication^17^. RNAi-mediated knockdown of ERI3 impaired accumulation of viral RNA and production of infectious particles for both DENV-2 and YFV17D, but not Enterovirus 71 (EV71). RNA immunoprecipitation of ERI3 from infected cells showed that ERI3 bound to DENV-2 and YFV17D RNA, with a clear preference for DENV-2 genomic RNA over sfRNA. ERI3 localized with Golgi markers, and this localization was driven by sequences in the N-terminus of ERI3. During DENV-2 infection, ERI3 relocalized, along with Golgi markers, to sites of DENV-2 replication. ERI3 was not required for the stability of viral RNA, but was required for DENV-2 RNA synthesis. The localization of ERI3 to the Golgi and its relocalization to DENV-2 replication sites during infection highlights a previously unappreciated role for the Golgi in viral replication.

## Results

### Proteomic screen for DENV-2 UTR-interacting proteins

**Figure 1:**
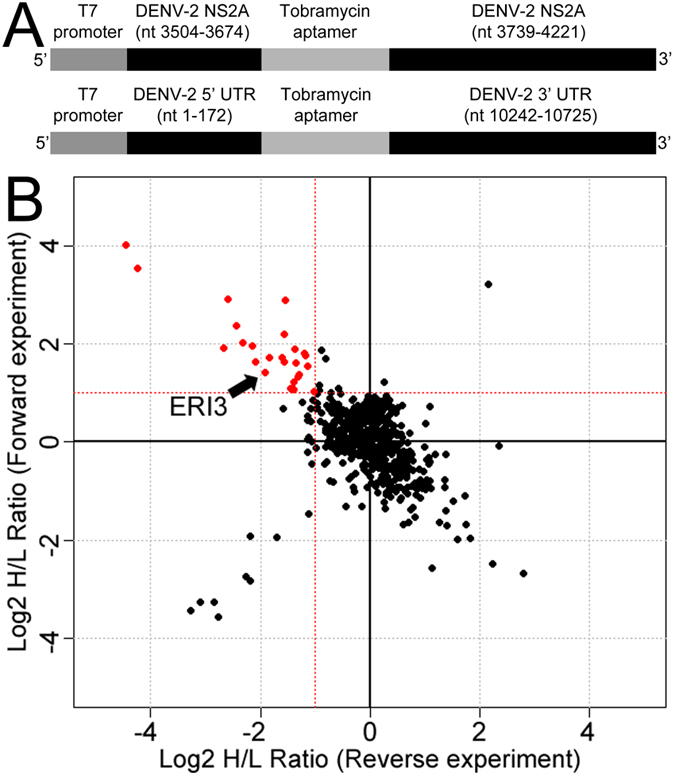
Identification of DENV-2 RNA interacting proteins. (**A**) Diagram of constructs used as T7 templates to generate RNA for chromatography. T7 promoter, aptamer sequence and nucleotide coordinates of sequences from DENV-2 NGC are indicated for the control NS2A and UTR constructs (figure adapted from^10^). (**B**) Scatter plot comparing the results from two independent quantitative mass spectrometric analyses of DENV-2 UTR-interacting proteins (three independent experiments were performed in total). In the experiment plotted on the y-axis (forward experiment), the control RNA was incubated with lysate from cells labeled with light arginine and lysine (K0R0) and the DENV-2 UTR RNA was incubated with lysate from cells labeled with heavy arginine and lysine (K8R10). In the experiment plotted on the x-axis (reverse experiment), the control RNA was incubated with heavy lysate and the DENV-2 UTR RNA was incubated with light lysate. The minimum of 2 or more fold change for both reverse and forward experiments were used to select the most confident interacting partners (red dots in upper left quadrant).

A previous study utilized HeLa cell lysates and RNA affinity chromatography coupled with quantitative mass spectrometry (qMS) to identify proteins that interact with the 5′ and 3′ untranslated regions (UTRs) of DENV-2 (New Guinea C strain)^10^,^12^. We performed a similar screen with HuH-7 cell lysates to identify proteins that bind to the DENV-2 UTRs in a commonly used cell culture model for *Flavivirus* replication. We defined DENV-2 UTR-interacting proteins as those enriched ≥2-fold over the control RNA in three independent experiments with at least ≥2 ratio counts. Thirty-seven (37) proteins met these criteria (Fig. 1 and Supplementary Table S1 “Pos strand RBPs”). Of these, twenty-one (21) proteins had been identified in our previous screen as candidate DENV-2 UTR-interacting proteins in HeLa cells^10^; among these 21 was the putative DEDD RNA exonuclease ERI3 (Fig. 1B and Supplementary Table S1 “New screen v Ward *et al*.^10^”). ERI3 was enriched for binding to the UTR RNA 2.6-fold, on average (SD = 0.255) (Supplementary Table S1). Indeed, ERI3 was independently identified as a candidate ‘hit’ in an RNAi screen for DENV-2 host factors in HuH-7 cells (Barrows *et al*., manuscript in preparation), suggesting that the binding we observed was functionally significant.

### ERI3 associates with DENV-2 and YFV17D, but not EV71 and MuV RNA

In order to validate and extend the role for ERI3 suggested by the proteomic screens, we utilized RNA immunoprecipitation and qPCR to test whether ERI3 interacts with DENV-2 RNA during infection. To determine if ERI3 interacts with other viral RNAs, we also performed RNA immunoprecipitation using cells infected with the *Flavivirus* YFV17D, the *Picornavirus* Enterovirus 71 (EV71) or the *Paramyxovirus* Mumps virus (MuV). We generated HEK293 cells expressing N-terminal FLAG-tagged ERI3 under the control of a tetracycline-inducible promoter. Expression of FLAG-ERI3 was induced and, 24 hours later, cells were infected with DENV-2 (MOI = 1.0), YFV17D (MOI = 1.0), EV71 (MOI = 0.3) or MuV (MOI = 1.0). 24 hours post-infection, lysates were harvested and immunoprecipitated using a mouse FLAG antibody or a mouse IgG control. Immunoprecipitated proteins were analyzed by western blot using antibodies to FLAG and the RNA binding protein DDX6. RNA precipitates were analyzed by qPCR for GAPDH and viral RNA.

**Figure 2:**
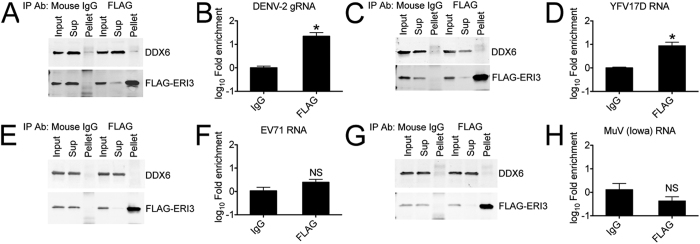
ERI3 associates with DENV-2 and YFV17D RNA *in vitro*. Flp-In T-Rex 293 cells with Tet-inducible FLAG-ERI3 expression were utilized for all RNA immunoprecipitations. Cells were treated with 5 μg/mL tetracycline for 24 hours, then infected with DENV-2 (**A**,**B**), YFV17D (**C**,**D**) or MuV (Iowa) (**G**,**H**) using a MOI = 1.0 or EV71 (**E**,**F**) using a MOI = 0.3. All infections and incubations were performed in the presence of 5 μg/mL tetracycline. 24 hours post-infection cells were harvested for RIP using 5 μg mouse IgG or mouse FLAG antibody. (**A,C,E**,**G**) Input, supernatant and pellet samples were assayed for the presence of DDX6 or FLAG-ERI3 by western blotting. (**B,D,F**,**H**) Input and pellet samples were assayed for the presence of the indicated viral RNA. Fold enrichment of viral RNA in the pellet was plotted relative to 1% of input RNA. Statistical significance was tested using a two-tailed T test. P values < 0.05 are indicated by an asterisk (*). Each RNA immunoprecipitation was performed in triplicate.

As seen in Fig. 2A,C,E,G, only FLAG antibody efficiently immunoprecipitated FLAG-ERI3 from lysates. We analyzed the input and pellet for viral RNA and determined that immunoprecipitation of FLAG-ERI3 significantly enriched DENV-2 genomic RNA (gRNA, 21.9-fold enrichment, P = 0.02) compared to the IgG control (Fig. 2B), demonstrating that ERI3 interacts with DENV-2 RNA during infection. To determine if FLAG-ERI3 immunoprecipitation preferentially enriched for gRNA or subgenomic flavivirus RNA (sfRNA), we performed qPCR using primers specific for sequence within the 3′ UTR and calculated the sfRNA concentration^18^. In contrast to gRNA alone, FLAG-ERI3 immunoprecipitation did not significantly enrich sfRNA (21.9-fold enrichment for gRNA, P = 0.02 vs. 0.45-fold enrichment for sfRNA, P = 0.09) (Fig. 2B and Supplementary Fig. S1A), suggesting that ERI3 preferentially binds to DENV-2 gRNA. Thus ERI3 is one of several factors identified by this RNA affinity chromatography method that have been shown to bind in infected cells and have functional importance^10^,^18^,^19^.

To determine whether ERI3 interacted with RNAs from other RNA viruses, we performed RNA immunoprecipitation and qPCR as described above using the YFV17D-, EV71- or MuV-infected cells expressing FLAG-ERI3. We analyzed the input and pellet using qPCR and determined that immunoprecipitation of FLAG-ERI3 significantly enriched YFV17D RNA (8.63-fold enrichment, P = 0.03) compared to the IgG control (Fig. 2D), suggesting there is an interaction between ERI3 and YFV17D RNA during infection. When we performed RNA immunoprecipitation using EV71- or MuV-infected cells expressing FLAG-ERI3 we determined there was a modest and not statistically significant enrichment of EV71 RNA in FLAG precipitates (EV71 = 2.45-fold enrichment, P = 0.05) and no enrichment for MuV (MuV = 0.426-fold enrichment, P = 0.85) (Fig. 2F,H). These results demonstrated that ERI3 preferentially associated with DENV-2 and YFV17D RNA compared to other viral RNAs such as EV71 or MuV and suggested a *Flavivirus*-specific function.

### ERI3 is required for DENV-2 and YFV17D, but not EV71, replication

RNA chromatography and immunoprecipitation experiments revealed an interaction between ERI3 and DENV-2 and YFV17D RNA, but not EV71 RNA (Figs 1 and 2). Additionally, ERI3 was a candidate ‘hit’ in an RNAi screen for DENV-2 host factors in HuH-7 cells (Barrows *et al*., manuscript in preparation). In order to test the functional importance of ERI3 for DENV-2 infection, we performed siRNA-mediated knockdown and infection with DENV-2, YFV17D or EV71. ERI3 knockdown was performed using two independent siRNAs (siERI3_III and siERI3_VII) transfected 48 hours prior to infection. As a positive control for knockdown of DENV-2 host factors, we performed siRNA-mediated knockdown of a subunit of the vacuolar ATPase (ATP6V0C) that is required for endosomal acidification and efficient virus replication^20^,^21^. At 24 hours post-infection, total cell lysates were harvested for western blot analysis or total cellular RNA was harvested and analyzed for viral RNA levels by qPCR. Tissue culture supernatants were harvested for foci forming assays or plaque assays.

**Figure 3:**
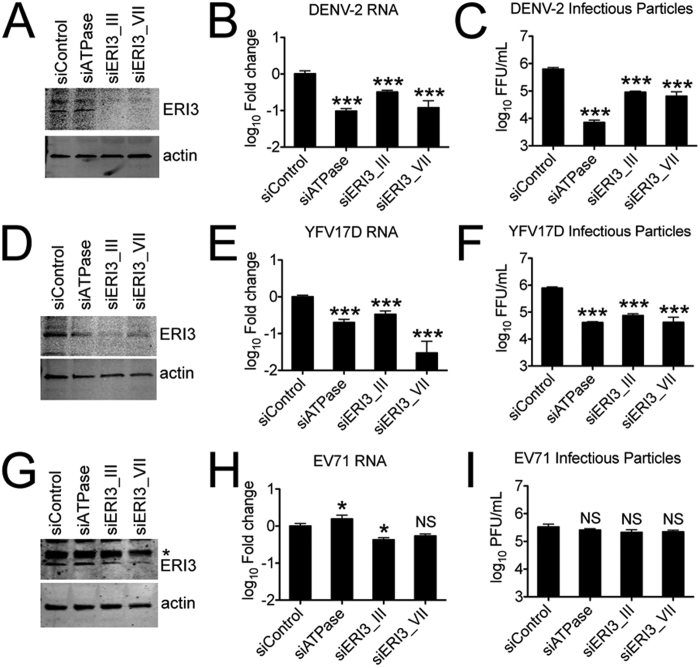
RNAi-mediated knockdown of ERI3 and infection with DENV2, YFV17D or EV71. HuH-7 or RD cells were transfected using 15 nM of the indicated siRNA and incubated for 48 hours. For DENV-2 and YFV17D infections, transfected HuH-7 cells were infected with an MOI = 1.0 and incubated 24 hours. For EV71 infection, transfected RD cells were infected with an MOI = 0.3 and incubated 24 hours. Cells and tissue culture supernatants were harvested and assayed for protein by western blot, RNA by quantitative real time PCR and infectious particle production by focus forming assay (DENV2 and YFV17D) or plaque assay (EV71). (**A,D**,**G)** Western blotting to test for knockdown of ERI3. Blots were probed with antibodies specific to actin or ERI3 and visualized using the Licor Odyssey Imaging system. (**B,E**,**H**) Quantitative real time PCR on total cellular RNA to assay for viral RNA content. Random cDNA was generated from total RNA and used in quantitative real time PCR using primers specific for GAPDH and DENV-2, YFV17D or EV71. Fold-change (∆∆Ct) relative to the control knockdown was calculated using GAPDH and viral RNA Ct values. Statistical significance was determined using a one-way ANOVA (CI = 95%) comparing samples to the siRNA control samples. (**C,F**,**I**) Focus or plaque assays for infectious particle production. Tissue culture supernatants from DENV-2 or YFV17D infections were used to inoculate BHK cell monolayers followed by overlay with CMC. Titer was determined by probing fixed cell monolayers using the pan-flaviviral Env antibody, 4G2 and anti-mouse DyLight680 secondary antibody and visualized using the Licor Odyssey Imaging system. Titer is expressed as focus forming units (FFU)/mL. Tissue culture supernatants from EV71 infection was used to inoculate Vero cell monolayers followed by overlay with CMC. Titer was determined by fixing the cell monolayers and staining with 1% Crystal Violet solution. Titer is expressed as plaque forming units (PFU)/mL. Statistical significance was determined using a one-way ANOVA (CI = 95%) comparing samples to the siRNA control samples. All knockdowns and infections were performed in triplicate in three independent experiments.

ERI3 knockdown was validated by western blot using an ERI3-specific antibody (Fig. 3A,D,G) and qPCR for ERI3 mRNA (Supplementary Fig. S2A–C). Knockdown of the vATPase subunit or ERI3 led to a 3- to 10-fold reduction in DENV-2 gRNA accumulation compared to the control knockdown (One-way ANOVA, P < 0.0001, Dunnett’s post-test P < 0.05) (Fig. 3B). Since the qPCR primers target the NS2A region of the viral genome and gRNA is incompletely degraded to generate sfRNA, we wanted to determine if gRNA was being degraded to sfRNA in the absence of ERI3. We performed qPCR using gRNA-specific primers and a set of primers that targets the 3′ UTR, which detects both gRNA and sfRNA. *In vitro* transcribed DENV-2 replicon RNA, containing both target sequences, was used to generate cDNA, perform qPCR and plot a standard curve for absolute quantitation of gRNA and sfRNA levels, as described in Bidet *et al*.^18^. Knockdown of the vATPase subunit led to a 10-fold reduction of the sfRNA:gRNA ratio compared to the control knockdown (One-way ANOVA, P < 0.021, Dunnett’s post-test, P < 0.05) (Supplementary Fig. S2D). DENV-2 replication is restricted early following knockdown of the vATPase subunit and RT-qPCR would primarily detect incoming viral genomes, leading to a reduction in the sfRNA:gRNA ratio (Fig. 3B; Supplementary Fig. S2D)^21^. Similarly, knockdown of ERI3 led to a 5- to 10-fold reduction of the sfRNA:gRNA ratio compared to the control knockdown (One-way ANOVA, P < 0.021, Dunnett’s post-test, P < 0.05) (Supplementary Fig. S2D). These results suggest that ERI3 is required for accumulation of both gRNA and sfRNA, and, therefore, an early process in replication is defective in the absence of ERI3.

Since ERI3 was shown to interact with YFV17D, but not significantly with EV71 viral RNA, we wanted to determine if ERI3 played a role in the accumulation of either YFV17D or EV71 RNA, similar to its role with DENV-2. Knockdown of the vATPase subunit or ERI3 led to a 3- to 90-fold reduction in YFV17D RNA accumulation compared to the control knockdown (One-way ANOVA, P < 0.0001, Dunnett’s post-test, P < 0.05) (Fig. 3E). In contrast, knockdown of the vATPase subunit or ERI3 led to a 0.7- to 2-fold reduction in EV71 RNA accumulation and statistical significance only with siATPase and siERI3_III (One-way ANOVA, P = 0.0012) (Fig. 3H). These results demonstrate that ERI3 is required for accumulation of DENV-2 and YFV17D gRNA, but not for EV71 RNA accumulation, suggesting a *Flavivirus*-specific function for ERI3.

Since knockdown of ERI3 reduced the accumulation of DENV-2 and YFV17D RNAs, we wanted to determine if ERI3 was required for production of infectious particles. Knockdown of the vATPase subunit led to a 19- to 88-fold reduction in YFV17D and DENV-2 infectious particle production, respectively, whereas ERI3 knockdown led to a 7- to 19-fold reduction in DENV-2 and YFV17D infectious particle production (One-way ANOVA, P < 0.0001, Dunnett’s post-test, P < 0.05) (Fig. 3C,F). In contrast, knockdown of the vATPase subunit or ERI3 did not lead to significant reduction of EV71 infectious particle production (One-way ANOVA, P = 0.0965) (Fig. 3I). These results suggest that ERI3 is required for DENV-2 and YFV17D, but not EV71, replication.

In addition, qPCR analysis of DENV-2 and YFV17D infection samples for expression of IFN-ß or IFITM2, an interferon-stimulated gene (ISG), showed that there was no significant difference in the levels of IFN-ß or IFITM2 expression in the absence of ERI3 (Supplementary Fig. S2E–H), suggesting ERI3 is required for a virus-specific function and not induction of the innate immune response. These results reveal that ERI3 is a host factor for both DENV-2 and YFV17D, but not EV71, and suggests a *Flavivirus*-specific role in replication in human cells.

DENV-2 infects both human and mosquitoes of the *Aedes* genus and we wondered whether the ERI3 requirement would be shared in these invertebrate hosts. We identified a single putative DEDD 3′–5′ exonuclease gene (AAEL007801) in *Aedes aegypti,* the major arthropod vector for DENV, and asked whether this gene was important for DENV-2 infection. *Aedes aegypti* mosquitoes were injected with double-stranded RNAs (dsRNAs) targeting AAEL007801 or GFP and then infected with DENV-2 via blood meal. Virus titers in mosquito midguts were then determined (Supplementary Fig. S3). Compared to the GFP control, dsRNA targeting AAEL007801 resulted in a modest (~30%) but statistically significant reduction in DENV-2 midgut titers, suggesting that AAEL007801, the mosquito homolog of ERI3, is also a mosquito host factor for DENV-2 (Supplementary Fig. S3 and Supplementary Table S2).

### ERI3 is not required for DENV-2 RNA stability but is required for DENV-2 RNA synthesis

**Figure 4:**
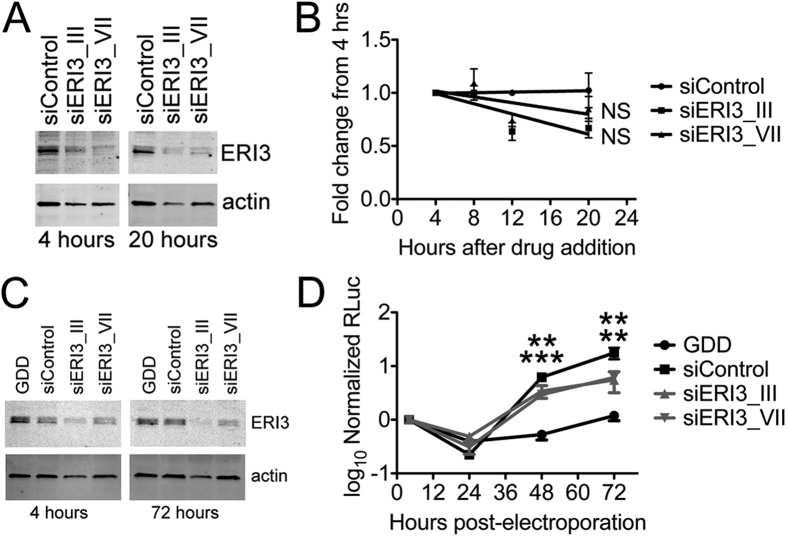
ERI3 is not required for DENV-2 RNA stability but is required for DENV-2 replicon function. HuH-7 cells were transfected with siRNAs targeting ERI3 and incubated for 48 hours prior to infection or electroporation. (**A**) Total cell lysates from control and ERI3 knockdown at 4 and 20 hours after addition of NITD008 were analyzed by western blot for ERI3 and pan-actin. (**B**) At 4, 8, 12 and 20 hours after addition of NITD008, total RNA from control and ERI3 knockdown was analyzed for DENV-2 RNA levels relative to cellular GAPDH RNA. Levels are expressed as the fold change from the 4 hour timepoint and were analyzed by linear regression using the GraphPad Prism software package. Three independent experiments were performed in triplicate. (**C**) Total cell lysates from control and ERI3 knockdown at 4 and 72 hours post-electroporation were analyzed by western blot for ERI3 and pan-actin. (**D**) Total cell were harvested at 4, 24, 48 and 72 hours post-electroporation and analyzed by dual luciferase assay for Renilla and firefly luciferase content. The GDD sample was electroporated with a mutant replicon that contains point mutations in the active site of NS5, rendering it replication deficient. For each timepoint, Renilla luciferase (RLuc) levels were normalized to the 4 hour firefly luciferase level. Normalized RLuc was plotted and analyzed by one-way ANOVA using the GraphPad Prism software package. Replicon assays were performed in three independent experiments.

ERI3 knockdown significantly reduced the accumulation of DENV-2 RNA and infectious virus at 24 hours post-infection and this was consistent with an effect on viral RNA replication. Given these observations we sought to define the role of ERI3 in DENV RNA replication. To determine if ERI3 functions in the synthesis or stability of DENV-2 RNA in infected cells, we developed an assay that would allow specific inhibition of virus transcription using the NS5 inhibitor NITD008 and analysis of DENV-2 RNA levels over time (Supplementary Information and Supplementary Fig. S4A,B)^22^. We performed siRNA-mediated knockdown of ERI3 followed by infection with DENV-2 as described above. Infected cells were harvested at 4, 8, 12 and 24 hours after addition of 25 μM NITD008 and assayed for DENV-2 RNA using qPCR. Levels of DENV-2 RNA were normalized to GAPDH and plotted as the fold-change from the 4-hour time point. A linear regression analysis was used to compare ERI3 knockdown to the control siRNA treatment. Western blot analysis confirmed ERI3 knockdown compared to the control siRNA and pan-actin loading control at the 4 and 20 hour time points (Fig. 4A).

The DENV-2 RNA appeared to be very stable, since the slope of the line in the control siRNA treatment is small (Slope = 0.00152 SD+/−0.000556) (Fig. 4B). Furthermore, while there was a modest reduction in DENV-2 RNA levels following NITD008 treatment in ERI3 knockdown cells, linear regression analysis determined there was no significant difference between the control siRNA treatment and knockdown with siERI3_III (Slope = −0.0239 SD +/−0.0121) or siERI3_VII (Slope = −0.136 SD +/−0.013) (P = 0.287). These results suggest that ERI3 does not function in maintaining the stability of DENV-2 RNA during replication.

Since ERI3 was not required for DENV-2 RNA stability, we tested whether ERI3 was required for synthesis of DENV-2 RNA. ERI3 knockdown was performed using two independent siRNAs (siERI3_III and siERI3_VII) transfected 48 hours prior to infection with DENV-2 (MOI = 1.0). As a control for inhibition of DENV-2 RNA synthesis, at 16 hours post-infection, NITD008 was added to a control infection. At 20 hours post-infection, total RNA was labeled for 30 minutes using 2 mM BrU and harvested for RNA immunoprecipitation using a BrU-specific antibody. In order to validate siRNA-mediated knockdown, we analyzed the input RNA by qPCR and determined that siRNA knockdown significantly reduced ERI3 mRNA levels (One-way ANOVA, P = 0.0001, Dunnett’s post-test, P < 0.05) (Supplementary Fig. S4C). Next, we analyzed the input and pellet for DENV-2 RNA and determined that NITD008 treatment led to a 5-fold reduction of BrU DENV-2 RNA relative to total input RNA (One-way ANOVA, P = 0.0013, Dunnett’s post-test, P < 0.05) (Supplementary Fig. S4D). Similarly, ERI3 knockdown led to a 3- to 5-fold reduction of BrU DENV-2 RNA relative to total input RNA, suggesting that ERI3 is required for DENV-2 RNA synthesis (One-way ANOVA, P = 0.0013, Dunnett’s post-test, P < 0.05) (Supplementary Fig. S4D).

To further substantiate a role for ERI3 in DENV-2 RNA synthesis, we tested the ability of a DENV-2 replicon to replicate in cells depleted of ERI3. The DENV-2 replicon has most of the structural protein coding sequence replaced with the Renilla luciferase (RLuc) ORF, but retains the non-structural protein coding sequences required for RNA synthesis^23^. The replicon RNA initiates translation and subsequently RNA replication when electroporated into cells, bypassing the need for receptor-mediated endocytosis. We transfected cells with a control siRNA or two independent siRNAs targeting ERI3 (siERI3_III and siERI3_VII), and electroporated cells with *in vitro* transcribed replicon RNA. To assay viral translation in the absence of RNA synthesis, we also used a replicon containing a mutation in NS5 that renders the RdRp inactive^24^. Cells were harvested at 4, 24, 48 and 72 hours post-electroporation and analyzed by western blot or dual-luciferase assay.

Western blots of cell lysates confirmed that ERI3 levels were reduced at 4 and 72 hours post-electroporation compared to the control siRNA knockdown (Fig. 4C). At 4 hours post-electroporation, there was no significant difference in RLuc levels with the NS5 mutant replicon or with the wildtype replicon in the control or ERI3 knockdown (Fig. 4D). This indicates that translation of the incoming replicon RNA is not impaired following ERI3 knockdown. We performed an independent polyribosome analysis of DENV-2-infected cells and found that GAPDH and DENV-2 RNA localize to similar fractions in control and ERI3 knockdown samples (Supplementary Fig. S4E–H), which was consistent with the replicon data.

When we examined the normalized Rluc values for ERI3 knockdown cells with the wild type replicon, we found that there was a significant difference in replication after knockdown at 48 (4-fold reduction) and 72 hours (6- to 8-fold reduction) post-electroporation compared to the control knockdown (One-way ANOVA, P = 0.0009, Dunnett’s post-test, P < 0.05) (Fig. 4D). While a translational effect only on progeny genomes cannot be ruled out, these data strongly suggest that ERI3 has a role in DENV-2 RNA synthesis.

### ERI3 localizes to COPII vesicles and Golgi structures

Since little is known about the cellular function of ERI3, we wanted to determine its subcellular localization. We tested the ERI3 antibody described above using indirect immunofluorescence microscopy on cells transfected with control or ERI3-specific siRNAs and determined that the antibody was not suitable for indirect immunofluorescence microscopy due to non-specific reactivity with nuclear components (data not shown). New polyclonal antibodies were raised against a recombinant GST-tagged N-terminal fragment of ERI3 (aa 1–135). To establish the specificity of the antibody to ERI3, we performed RNAi-mediated knockdown of ERI3 in HuH-7 cells and probed with antibodies for ER markers (HSPA5 or CANX) and ERI3. We analyzed knockdown by western blot (Supplementary Fig. S5A) and confocal microscopy (Supplementary Fig. S5B,C), and determined that ERI3 signal was reduced in knockdown cells compared to control knockdown (Supplementary Fig. S5A–C).

**Figure 5:**
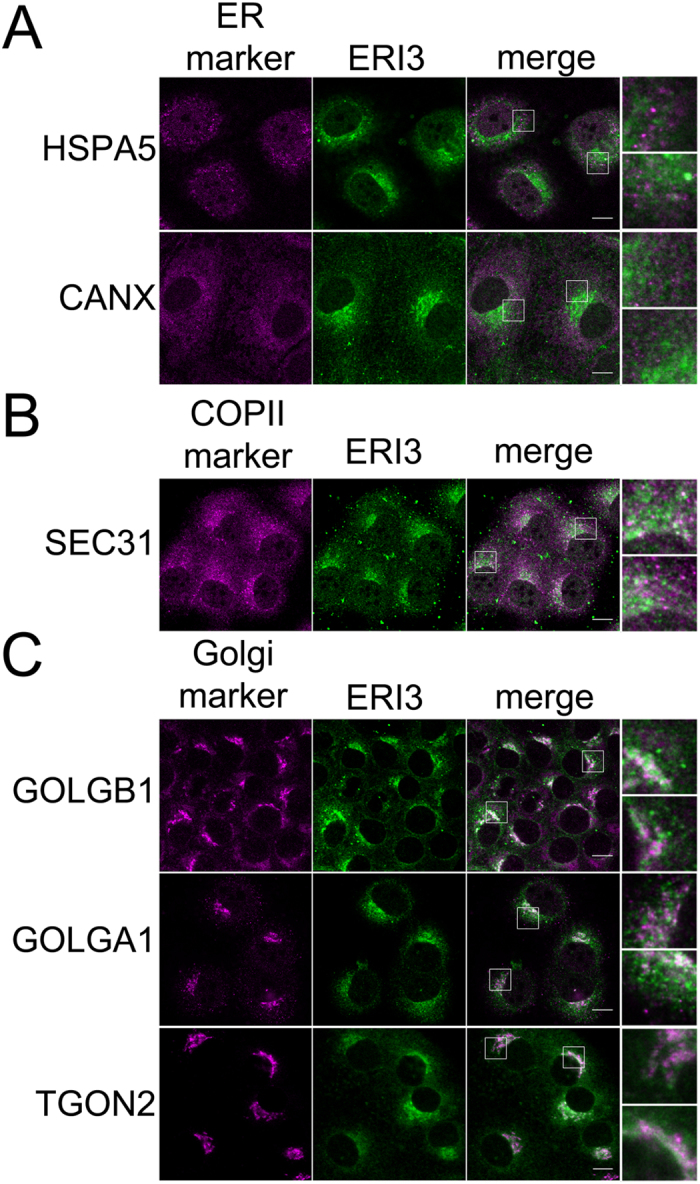
ERI3 localizes to Golgi structures and COPII vesicles. HuH-7 cells were plated on coverslips and fixed, permeabilized and probed with antibody to ERI3 and the indicated organellar marker protein. Fluorescent confocal micrographs show (from left to right) organellar marker, ERI3, overlay, and insets from the indicated region in the overlay panel. (**A**) Localization of ERI3 and the ER markers HSPA5 and CANX. (**B**) Localization of ERI3 and the COPII marker Sec31. (**C**) Localization of ERI3 and the Golgi markers GOLGB1, GOLGA1 and TGON2. Scale bar = 10 μm. Localization was analyzed in at least three independent experiments.

To determine the localization of ERI3, we probed HuH-7 cells with antibody to ERI3 and antibodies to organelle markers from the endoplasmic reticulum (ER) (HSPA5 (also BiP, GRP78) and CANX), COPII vesicles (SEC31) or Golgi (GOLGB1 (also Giantin), GOLGA1 (also Golgin-97) and TGON2 (also TGN46)). The staining pattern for ERI3 did not coincide with the ER markers HSPA5 or CANX (Fig. 5A). In contrast, the COPII marker SEC31 had a partially overlapping staining pattern with ERI3 (Fig. 5B). Interestingly, the Golgi markers GOLGB1, GOLGA1 and TGON2 all exhibited a similar staining pattern as ERI3 (Fig. 5C), suggesting that ERI3 localizes in close proximity to the Golgi in uninfected cells. Additional studies using ERI3 sequences fused to EGFP suggest that Golgi localization is driven by sequences in the N-terminal portion of ERI3 (Supplementary Information and Supplementary Fig. S6). Taken together, these results suggest that ERI3 resides in close proximity to the Golgi and its localization is driven by sequences in the N-terminus.

### ERI3 and TGON2 relocate to sites of DENV-2 replication during infection

**Figure 6:**
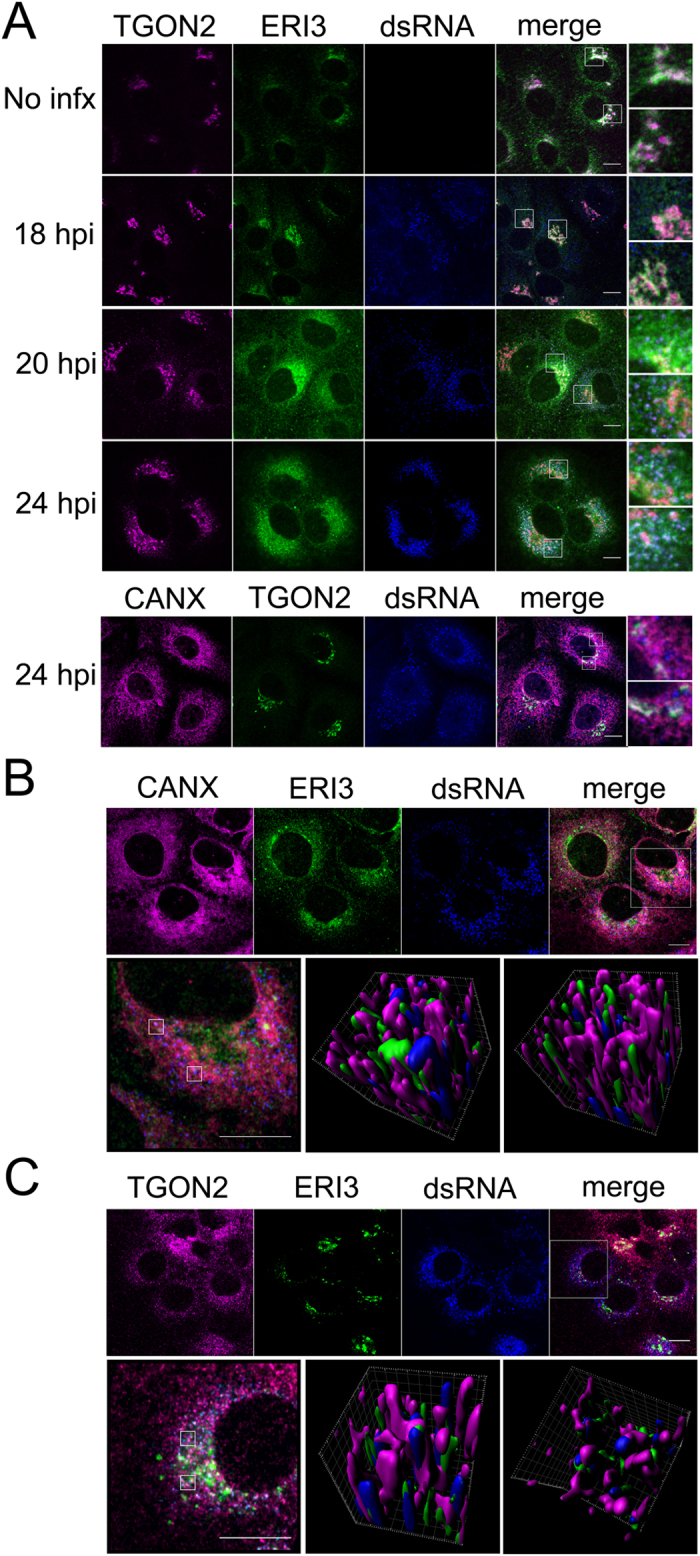
ERI3 relocalizes to viral replication sites in DENV-2-infected cells. HuH-7 cells were plated on coverslips and infected with DENV-2. At the indicated time post-infection, cells were fixed, permeabilized and probed with antibodies. Fluorescent confocal micrographs show (from left to right) ERI3, dsRNA, the indicated organellar marker, overlay, and insets from the indicated region in the overlay panel. (**A**) Coverslips from different timepoints post-infection were probed with antibodies to ERI3, dsRNA and either the Golgi marker TGON2 or the ER marker CANX. (**B**,**C**) Coverslips from 24 hour DENV-2 infections were probed with antibodies to ERI3, dsRNA and the ER marker CANX or Golgi marker TGON2. Three-dimensional rendering of the regions identified in the insets were generated using the Imaris software package. Localization was analyzed in at least three independent experiments.

Our data demonstrated that ERI3 interacts with DENV-2 RNA (Figs 1 and 2) and is required for DENV-2 replication and RNA synthesis (Figs 3 and 4). Paradoxically, DENV-2 replication has been shown to take place in the ER^6^,^7^, whereas ERI3 has been shown to localize to Golgi structures (Fig. 5 and Supplementary Fig. S6). In order to determine the localization of ERI3 during DENV-2 infection, we infected HuH-7 cells for 18, 20 or 24 hours and probed with antibodies to ERI3, TGON2 and dsRNA, a marker for DENV-2 replication sites. As seen in Fig. 6, ERI3 and TGON2 localized to condensed cytoplasmic structures, consistent with the Golgi in uninfected cells (Fig. 6A). At 18 hours post-infection, ERI3 primarily localized to similar condensed Golgi structures as TGON2, distinct from areas marked by anti-dsRNA (Fig. 6A). At 20 and 24 hours post-infection, however, the condensed Golgi structure appeared to expand or break down and both ERI3 and TGON2 relocated to within close proximity of dsRNA (Fig. 6A). To confirm that TGON2 was relocalizing to ER-derived replication sites, we probed with antibodies to TGON2, dsRNA and the ER marker CANX. At 24 hours post-infection, TGON2 and CANX localized near to dsRNA, confirming that TGON2 is relocalizing to similar sites as CANX (Fig. 6A). These data demonstrate that ERI3, along with Golgi-associated membranes, are found near sites of DENV-2 replication during infection. The localization of ERI3 in infected cells and its role in RNA synthesis probably explains why we do not detect significant interactions with the DENV-2 sfRNA.

DENV-2 viral replication sites are subdiffraction-limited, and thus determining their precise localization and milieu within the cell is challenging using traditional light microscopy methods. In order to further resolve the structures formed by ERI3, dsRNA and ER (CANX) or Golgi markers (TGON2) at replication sites, we performed additional imaging by three-dimensional super resolution microscopy. At 24 hours post-infection, samples were prepared as described previously and imaged using the Zeiss LSM880 confocal, equipped with an Airyscan detector capable of 140 nm resolution. DENV-2 infected cells were identified by positive staining for dsRNA and, consistent with the previous results (Fig. 6A), ERI3 relocalized to the vicinity of replication sites as punctate aggregates (Fig. 6B). CANX, a previously described marker for replication sites^6^, localized to the ER and nuclear envelope and near to viral replication sites. Volumetric rendering of these sites elucidated microdomains containing small membranous sheets of CANX along with dsRNA and ERI3 (Fig. 6B). We performed a similar analysis using samples stained for dsRNA, ERI3 and TGON2. TGON2 relocalized from the Golgi and formed interconnected membranous structures surrounding the ERI3 puncta and replication sites, and dsRNA, ERI3 and TGON2 were observed in close proximity to one another without direct colocalization observed (Fig. 6C). These results demonstrate that ERI3, as well as ER and Golgi markers, relocalize to replication sites in DENV-2 infected cells. Interestingly, these results suggest that both the ER and Golgi membranes are being remodeled in DENV-2-infected cells.

## Discussion

Utilizing RNA chromatography coupled with quantitative mass spectrometry, we identified cellular proteins that interact with the DENV-2 UTRs. ERI3 bound preferentially to DENV-2 gRNA likely through an interaction with the UTRs, where. several conserved secondary structures have been identified in *Flaviviruses*^11^,^24^,^25^,^26^. We propose here that ERI3 is a host factor that interacts with the viral UTRs and specifically regulates DENV-2 RNA synthesis, but not translation.

RNAi knockdown studies demonstrated that ERI3 is required for DENV-2 replication in both human cells and mosquitoes (Fig. 3 and Supplemental Fig. S3). Additional studies support a role for ERI3 in DENV-2 and YFV17D, but not EV71, replication in human cells (Fig. 3). Furthermore, RNA immunoprecipitation data established that ERI3 interacts with YFV17D RNA, though ERI3 was not identified in a RNA chromatography screen for YFV17D-interacting proteins (Fig. 2C,D and data not shown). Since EV71 replication did not require ERI3, it is possible that ERI3 is a *Flavivirus*-specific host factor, and not a positive-strand RNA virus host factor (Fig. 3G–I). However, additional experiments would be required to test this.

ERI3 is likely a 3′–5′ exonuclease given its homology to ERI1 and preliminary experiments we have carried out in collaboration with Drs W. Marzluff and Z. Dominski (UNC, Chapel Hill). Several studies have established roles for 3′–5′ RNA exonucleases in viral replication. Lassa fever virus nucleoprotein has been shown to possess dsRNA-specific 3′–5′ exonuclease activity that degrades dsRNA replication intermediates that can stimulate the host innate immune response^27^. Our studies suggest that ERI3 function in viral replication is not linked to the host innate immune response, since RT-qPCR analysis showed similar levels of IFN and ISG mRNA expression following infection in control and ERI3 knockdown cells (S2 Fig. E–H). Coronavirus nsp14 has been shown to possess 3′–5′ exonuclease activity that is required for maintaining replication fidelity of the viral RdRp^28^,^29^,^30^. Since DENV-2 has been shown to undergo 3′ end repair, it is possible that ERI3 is required for excision of non-templated nucleotides and maintenance of genome fidelity^31^. However, mutations that inactivate the proofreading activity of Coronavirus nsp14 had no effect on replication kinetics in tissue culture, suggesting that viral RNA synthesis and fidelity are not necessarily linked^30^. Moreover, our preliminary data suggest that exonuclease activity is not required for the pro-viral action of ERI3 (data not shown). While additional experiments are required to define the precise function in DENV-2 RNA synthesis, ERI3 could be an attractive host factor for therapeutic targeting in both humans and mosquitoes.

Our most unexpected data showed that ERI3 localized to Golgi structures in uninfected cells, a process driven by N-terminal amino acid sequences. However, in DENV-2-infected cells, ERI3 and the Golgi resident protein TGON2 relocalized near to sites of viral replication. Super-resolution imaging by Airyscan revealed that ERI3, as well as ER and Golgi markers, localized near viral replication sites, suggesting DENV-2 induces relocalization of these proteins to facilitate virus replication. *Flaviviruses* induce extensive membrane rearrangements during infection, and previous studies using DENV-2 implicated primarily ER-derived membranes^6^,^7^. In contrast, studies of membrane rearrangements induced by the Kunjin strain of WNV suggest that Golgi proteins are recruited to replication sites^8^,^9^. Furthermore, Brefeldin A inhibition of Golgi-ER retrograde transport has been shown to inhibit Kunjin virus replication, suggesting a critical role for Golgi proteins in *Flavivirus* replication^32^. Here, we present evidence that, in addition to ER-resident proteins, DENV-2 recruits Golgi-resident proteins to replication sites, highlighting a possible role for the Golgi in establishment or maintenance of viral replication. One model implicating Golgi proteins in DENV-2 replication is that they are required to establish replication sites in the ER membrane, either by initiating or stabilizing novel membrane structures in the ER. Indeed, several host factors, including Golgi-associated proteins, have been identified that mediate membrane deformation and establishment of DENV and other positive strand virus replication sites, including FASN and GBF1^33^. However, it is not clear whether ERI3 provides a functional link between membrane remodeling and viral RNA synthesis or is simply recruited from the Golgi to replication sites for synthesis-specific functions.

In summary, we have identified a novel putative 3′–5′ RNA exonuclease and critical DENV-2 host factor in humans and mosquitoes that associates with the DENV-2 UTR and is required for RNA synthesis and productive viral replication. Relocalization of ERI3 and other Golgi proteins to DENV-2 replication sites reveals a previously unknown function for the Golgi in Dengue virus replication.

## Methods

### Cell lines and viruses

HuH-7 (Duke Cell Repository), RD (American Tissue Culture Collection CCL-136) and Vero (Duke Cell Repository) cells were maintained in DMEM (Life Technologies) supplemented with 10% FBS (HyClone) and 0.5 μg/mL gentamicin sulfate (Life Technologies). BHK-21 cells (a kind gift from Dr. Eng Eong Ooi, Duke-NUS, Singapore) were maintained in RPMI (Life Technologies) supplemented with 10% FBS and 0.5 μg/mL gentamicin sulfate (Life Technologies). For SILAC labeling, HuH-7 cells were maintained as described in Ward *et al*.^12^. Flp-In T-Rex 293 cells^34^ expressing N-terminal FLAG-tagged full-length ERI3 were generated by cotransfection of pcDNA5FRT/TO-FLAG-ERI3 and pOG44 (expressing Flp recombinase) and selection for blasticidin (10 μg/mL) and hygromycin resistance (200 μg/mL)^35^. For *Flavivirus* infection, cells were infected with DENV-2 NGC or YFV17D (kind gifts from Eng Eong Ooi, Duke-NUS) in serum-free DMEM for 1 hour using a MOI of 1.0. For EV71 infection, cells were infected with Enterovirus 71 Strain 41 in serum-free DMEM for 1 hour using a MOI of 0.3. For MuV infection, cells were infected with MuV (Iowa) (a kind gift from Biao He, University of Georgia) in serum-free DMEM for 1 hour using an MOI of 1.0. Inoculum was replaced with fresh media and incubated for 24 hours prior to analysis.

For mosquito infection assay, C6/36 cells (*Aedes albopictus*, American Tissue Culture Collection CRL-1660) were used to produce DENV-2 NGC stocks. C6/36 cells were maintained in minimal essential medium (MEM, Gibco) with 10% heat inactivated FBS, 1% L-glutamine, 1% penicillin-streptomycin, and non-essential amino acids at 32 °C with 5% CO_2_. DENV-2 NGC stocks were produced in C6/36 cells grown at 80% confluence. Cells were infected at a multiplicity of infection (MOI) of 0.5 and the infection was allowed to proceed for 144 h before collection of supernatant.

### RNA chromatography and quantitative mass spectrometry

RNA chromatography and quantitative mass spectrometry analysis was performed as described in Ward *et al*.^12^. For binding site mapping, RNA chromatography was performed as above, but samples were analyzed by western blot for ERI3, DDX6 and streptavidin as described in Bidet *et al*.^18^.

### RNA immunoprecipitation

Flp-In T-Rex 293 cells expressing N-terminal FLAG-tagged full-length ERI3 (GenBank NP_076971.1) were generated as described above. 24 hours prior to infection, fresh DMEM containing 10% FBS, 0.5 μg/mL gentamicin sulfate and 5 μg/mL tetracycline was added to the cells to induce FLAG-ERI3 expression. Cells were infected as described above and incubated for an additional 24 hours prior to harvesting for RNA immunoprecipitation. RNA immunoprecipitation was performed using 5 μg of mouse IgG or FLAG MS2 antibody (Sigma) and the MagnaRIP kit according to the manufacturer’s protocol (Millipore). Input and pellet fractions were analyzed by western blot for FLAG, DDX6 and pan-actin proteins and by SybrGreen qPCR for GAPDH and viral RNA (qPCR described below). Relative levels of viral RNA from each pellet were calculated using the fold-enrichment method described in the Imprint RIP kit (Sigma) and analyzed by two-tailed t-test using the GraphPad Prism software package.

### RNAi-mediated knockdown in human cells

For all siRNA-mediated knockdown experiments, cells were reverse transfected according to manufacturer’s instructions using RNAiMAX (Life Technologies) and a final concentration of 15 nM siRNA (Singapore Advanced Biologics). Sequences of all siRNAs used in this study are listed in Supplementary Table S3. Cells were incubated for 48 hours and infected with virus or electroporated with replicon RNA.

### Western blots

Samples were separated by SDS-PAGE (BioRad), transferred to Immobilon FL (Millipore) and probed with indicated antibodies in PBS containing 5% milk and 0.5% Tween 20. Blots were washed with 0.5% Tween 20 in PBS and primary antibodies were detected by incubating with anti-rabbit DyLight800 and anti-mouse DyLight680 antibodies. Blots were washed in 0.5% Tween 20 in PBS and a final wash was performed in PBS prior to imaging using the Odyssey infrared imaging system (LiCor).

### Quantitative PCR

Total RNA was extracted from cells using the EZNA Total RNA kit (Omega Bio-Tek) and cDNA was synthesized using iScript (BioRad) according to the manufacturers’ instructions. Levels of cDNA were analyzed by SybrGreen real time PCR (BioRad) using target-specific primers listed in S3 Table. Levels of each RNA were calculated relative to GAPDH using the ∆∆Ct method and analyzed by One-way ANOVA and Dunnett’s post-test using the GraphPad Prism software package. For absolute quantitation and calculation of the sfRNA:gRNA ratio, 1 ng of replicon RNA was serially diluted 10-fold and used to generate a cDNA standard curve. Ct values from gRNA and sfRNA + gRNA real time PCR were plotted on the standard curve to generate absolute molecules of RNA for each species. The final sfRNA:gRNA ratio was plotted and analyzed by One-way ANOVA and Dunnett’s post-test using the GraphPad Prism software package.

### Focus assay

Focus assays for DENV-2 and YFV17D were performed as described in Ward *et al*.^10^ using the pan-*Flavivirus* anti-Env 4G2 monoclonal antibody^10^.

### Plaque assay

Plaque assay for EV71 infectious particle production was performed using 10^5^ Vero cells plated in 24-well plates. 24 hours later, cell monolayers were washed with serum-free DMEM and overlaid with 10-fold dilutions of tissue culture supernatant from EV71 infections. After one hour, inoculum was replaced, cells were washed with serum-free DMEM and overlaid with 1% CMC containing DMEM and 2% FBS. 72 hours later, overlay media was removed and cells were fixed for 15 minutes with 1XPBS containing 3.75% formaldehyde. Fixed cells were stained for 30 minutes using 1XPBS, 3.75% formaldehyde, 10% Crystal Violet and washed to visualize plaques. Plaques were counted, expressed as pfu/mL of inoculum and analyzed by One-way ANOVA and Dunnett’s post-test using the GraphPad Prism software package.

### Mosquito infections

*Aedes aegypti* Rockefeller/UGAL strain mosquitoes were maintained on a 10% sucrose solution at 27 °C and 95% humidity with a 12-hr light/dark cycle as described previously^36^.

For the silencing assay of mosquito exonuclease, dsRNAs targeting AAEL007801, or GFP as a control, were synthesized using the HiScribe T7 *in vitro* transcription kit (New England Biolabs). The primer sequences used for dsRNA synthesis, as well as those used to confirm gene silencing by real-time PCR are listed in Table S3. dsRNA was injected into the thorax of cold-anesthetized 6-day-old female mosquitoes using a nano-injector (Nanoject; Drummond Scientific) with a glass capillary needle. Control mosquitoes were injected with an equal amount of dsRNA targeting GFP. Injected mosquitoes were incubated for 3 days prior to DENV infection through blood feeding^36^,^37^.

Virus stock (DENV-2 NGC) was mixed 1:1 with commercial human blood supplemented with 10% human serum and 1% 100 mM ATP (Thermo Scientific). The infectious blood meal was maintained at 37 °C for 30 min and then offered to dsRNA-injected mosquitoes via an artificial membrane feeding system. The blood-fed mosquitoes were maintained in the insectary under the conditions mentioned above for 7 days prior to midgut dissection and determination of infection phenotype. The dissection was performed in 1 drop of 1x PBS, and the dissected midguts were transferred to a microcentrifuge tube containing 150 μl of MEM and stored at −80 °C until used for virus titration. DENV-2 titers in midguts were determined by plaque assay on BHK-21 cells as described above.

### Statistical analyses of mosquito infection data

The Mann-Whitney U-test was used to compare the midgut DENV titers of the control group and experimental groups. Statistical analyses were conducted using the GraphPad Prism 5.05 statistical software package. Statistical significance is indicated with asterisks: **p < 0.01. Descriptive statistics for DENV infection assays are presented in Supplementary Table S2.

### Analysis of DENV-2 RNA stability

ERI3 knockdown was performed as described above using two independent siRNAs (siERI3_III and siERI3_VII) transfected 48 hours prior to infection. Cells were infected with DENV-2 using an MOI of 1.0 and incubated for 16 hours before addition of NITD008 (Novartis Institute of Tropical Diseases, 25 μM final concentration). Total RNA was harvested and analyzed for DENV-2 RNA levels relative to GAPDH by qPCR as described above. Each time point was calculated relative to the 4 hour time point and linear regression analysis was performed using GraphPad Prism.

### Analysis of DENV-2 RNA synthesis

ERI3 knockdown was performed as described above using two independent siRNAs (siERI3_III and siERI3_VII) transfected 48 hours prior to infection. Cells were infected with DENV-2 using an MOI of 1.0 and incubated for 16 hours before addition of NITD008 (25 μM final concentration). Four hours after drug addition, infected cells were pulse-labeled with 2 mM BrU (Sigma) for 30 minutes and harvested for total RNA^38^. Nascent transcripts were immunoprecipitated using a BrU-specific antibody (US Biologicals) and quantified by qPCR for GAPDH or DENV-2 RNA. BrU RNA levels were normalized relative to input RNA and analyzed by One-way ANOVA and Dunnett’s post-test using the GraphPad Prism software package.

### Polyribosome analysis

Polyribosome analysis was performed as described in Bidet *et al*.^18^.

### Replicon assay

HuH-7 cells were transfected with siRNA, as described above, 48 hours prior to electroporation with the DENV-2 replicon (a kind gift from Eva Harris, UC, Berkeley)^23^. Replicon assay was performed as described in Bidet *et al*.^18^.

### Confocal immunofluorescence microscopy

HuH-7 cells were plated on 22 × 22 mm #1.5 coverslips. For uninfected coverslips, 24 hours later, cells were washed two times with ice cold PBS and fixed for 10 minutes at room temperature in 1XPBS/3.75% formaldehyde followed by permeabilization for 10 minutes in 1XPBS/0.1% Triton X-100. For infected coverslips, cells were infected with DENV-2 NGC at an MOI of 10, incubated for 24 hours, then fixed and permeabilized as above. Following permeabilization, coverslips were washed two times with PBS and stored at 4 °C. Coverslips were probed with indicated antibody in PBST (1XPBS, 0.05% Tween-20), washed 2X with PBST and incubated with fluorescent secondary antibody in PBST. After washing 2X with PBST and 1X with PBS, coverslips were mounted on slides using ProLong Gold (Life Technologies). Primary antibodies were obtained from various manufacturers (listed in Supplementary Table S3) and secondary antibodies were obtained from Life Technologies. Samples were imaged on a Leica white light laser TCS-SP8 inverted laser scanning confocal microscope, using an HC PL APO CS2 63x/1.40 oil immersion objective. For acquiring the super resolution images, these same samples were imaged on a Zeiss LSM 880 with an Airyscan detector using a PL-APO 63x/1.4 M27 oil immersion objective. 11 confocal Z-slices were acquired every 180 nm and the resulting Z-stack was reconstructed and rendered in three dimensions using Imaris software (Bitplane).

## Additional Information

**How to cite this article**: Ward, A. M. *et al*. The Golgi associated ERI3 is a *Flavivirus* host factor. *Sci. Rep.* **6**, 34379; doi: 10.1038/srep34379 (2016).

## Supplementary Material

Supplementary Information

Supplementary Dataset 1

Supplementary Dataset 2

Supplementary Dataset 3
