# Supplementary material for: The Golgi associated ERI3 is a Flavivirus host factor
Source: Sci Rep. 2016 Sep 29;6:34379. doi: 10.1038/srep34379 (PMC5041148; doi:10.1038/srep34379)
Supplement: Supplementary Information [file srep34379-s1.pdf]

Supplementary Information

The Golgi associated ERI3 is a *Flavivirus*-specific host factor for Dengue virus

Alex Michael Ward[1]*, Meredith E.K. Calvert[2,#a], Leah R. Read[1,#c], Seokyoung Kang[4], Brandt E. Levitt[3], George Dimopoulos[4], Shelton S. Bradrick[3,5], Jayantha Gunaratne[6], Mariano A. Garcia-Blanco[1,3,5,7]*

[1]Program in Emerging Infectious Diseases, Duke-NUS Graduate Medical School, Singapore

[2]Bioimaging & Biocomputing Facility, Temasek Lifesciences Laboratory, Singapore

[3]Center for RNA Biology and Department of Molecular Genetics and Microbiology, Duke University Medical Center, Durham, North Carolina, United States of America

[4]W. Harry Feinstone Department of Molecular Microbiology and Immunology, Bloomberg School of Public Health, Johns Hopkins University, Baltimore, Maryland, United States of America

[5]Department of Biochemistry and Molecular Biology, The University of Texas Medical Branch, Galveston, Texas, United States of America

[6]Mass Spectrometry and Systems Biology Laboratory, Institute of Molecular and Cell Biology, Singapore

[7]Department of Medicine, Duke University Medical Center, Durham, North Carolina, United States of America
[#a]Current address: Histology and Light Microscopy Core, Gladstone Institutes, San Francisco, California, United States of America
[#c]Current address: Ontario Veterinary College, University of Guelph, Guelph, Ontario, Canada
*Corresponding authors: alex.ward@duke-nus.edu.sg (AMW) and mariano.garciablanco@duke-nus.edu.sg (MAG-B)

Validation of DENV-2 RNA stability and synthesis assay

To validate the assay, we tested the ability of DENV-2-infected cells to incorporate BrU into viral RNA in the presence or absence of the adenosine analog and DENV-2 NS5 inhibitor NITD008[1]. For the stability and synthesis assays, ERI3 knockdown was performed using two independent siRNAs (siERI3_III and siERI3_VII) transfected 48 hours prior to infection. Cells were infected with DENV-2 using an MOI of 1.0 and incubated for 16 hours before addition of NITD008 (25 μM final concentration). For the stability assay, time points were harvested as described above. For the synthesis assay, four hours after drug addition, infected cells were pulse-labeled with 2 mM BrU for 30 minutes and harvested for total RNA[2]. Nascent transcripts were immunoprecipitated using a BrU-specific antibody and quantified by qPCR for GAPDH or DENV-2 RNA. As shown in S4 Figure A, the BrU antibody significantly enriched for GAPDH transcripts compared to the IgG control with or without the addition of NITD008. In addition, there was no significant difference in enrichment for GAPDH RNA with or without NITD008 (Tukey's post-test, NS). The BrU antibody significantly enriched for DENV-2 transcripts in the absence of NITD008 compared to the IgG control (One-way ANOVA, $F(2,6)=37.78$, $P=0.0004$, Tukey's post-test, $P<0.05$), but there was no significant difference between the IgG control and BrU immunoprecipitation in the presence of NITD008 (Tukey's post-test, NS) (S4 Fig. A and B). Altogether, these results demonstrate that NITD008 specifically inhibits DENV-2 but not GAPDH transcription at 4 hours post-addition.

Sequences in the N-terminus target ERI3 to Golgi structures

Since native ERI3 was shown to localize near to the Golgi, we wanted to determine whether Golgi targeting was specified by sequences in ERI3. We tested a series of EGFP-ERI3 fusion proteins for their ability to colocalize with the Golgi marker TGON2; EGFP fusion constructs were generated that contain the full-length ERI3 sequence (aa 1-337), the C-terminal exonuclease domain (aa 136-337), or N-terminal sequences (aa 1-135 and aa 1-44) (S5 Fig. A). HuH-7 cells were transfected with EGFP fusion constructs and probed with antibodies to EGFP, ERI3 and TGON2. EGFP fusion protein containing full-length ERI3 localized throughout the cell and colocalized with TGON2 (S5 Fig. B). In contrast, EGFP fused to ERI3 aa 136-337 localized primarily to the nucleus and showed no overlap with native ERI3 or TGON2 (S5 Fig. B). It should be noted that the ERI3 antibody was generated using aa 1-135, therefore proteins lacking these sequences are not detected. In contrast, EGFP fused to ERI3 aa 1-135 localized primarily to the cytoplasm and had a similar localization pattern as native ERI3 and TGON2 (S5 Fig. B). The localization to the Golgi structures was abolished in a more abbreviated C-terminal truncation in which EGFP was fused to ERI3 aa 1-44, which had a diffuse staining pattern in the cell (S5 Fig. B). These data suggest that N-terminal sequences program ERI3 for localization to Golgi structures.

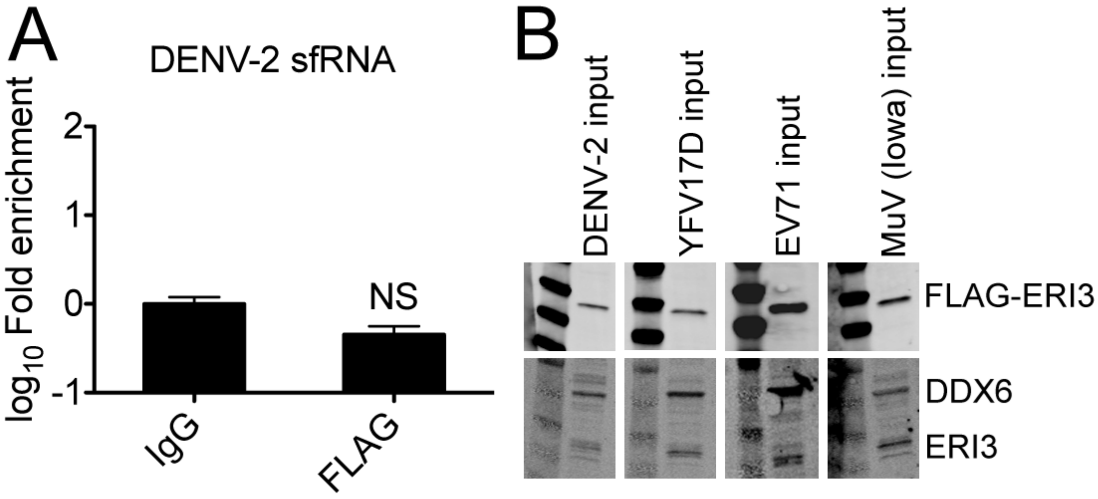

Supplementary Figure S1: Analysis of ERI3 RNA immunoprecipitation. (**A**) ERI3 RNA immunoprecipiation does not significantly enrich for DENV-2 sfRNA. Pellet fractions were analyzed for the presence of gRNA and sfRNA using oligos specific for sequences in the 3' UTR of DENV-2. Fold enrichment was calculated and analyzed by paired t-test. (**B**) Expression of FLAG-tagged ERI3 and endogenous ERI3 in input samples from RNA immunoprecipitation experiments. Lysates were analyzed for FLAG-ERI3 and native ERI3 expression by western blot for FLAG, ERI3 and DDX6.

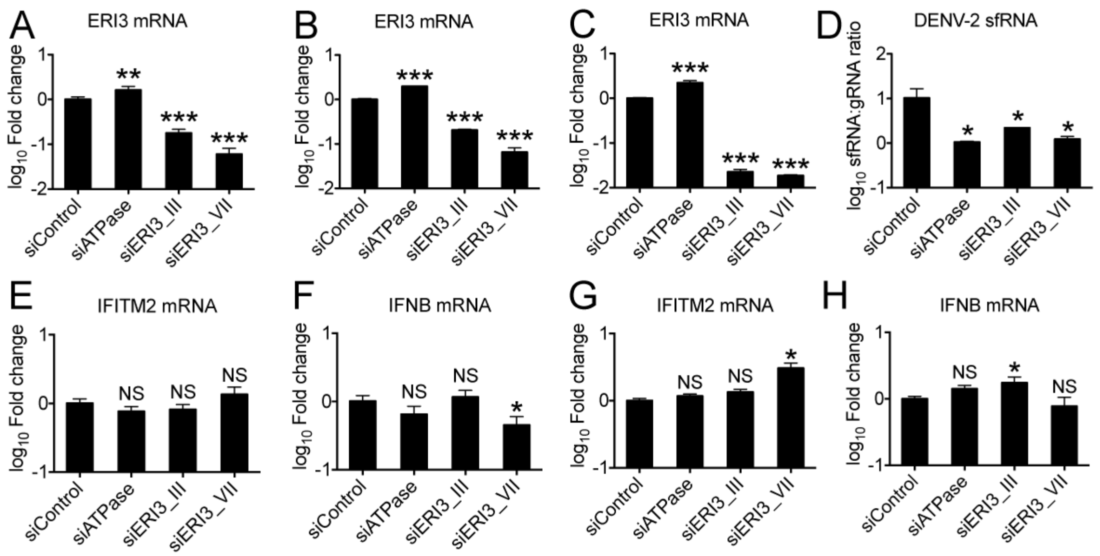

Supplementary Figure S2: Effect of ERI3 knockdown on viral replication. (**A**, **B** and **C**) Analysis of ERI3 mRNA knockdown. Total cellular RNA from knockdown and infection with DENV-2, YFV17D or EV71 was analyzed by qPCR for ERI3 mRNA levels normalized to GAPDH mRNA. ERI3 mRNA levels relative to the control knockdown was calculated and analyzed by one-way ANOVA using the GraphPad Prism software package. (**D**) Analysis of sfRNA levels following ERI3 knockdown and DENV-2 infection. Total cellular RNA from knockdown and infection was analyzed using primer sets specific for gRNA or gRNA and sfRNA. Absolute quantification of gRNA and sfRNA levels was determined by comparison to a standard curve of *in vitro* transcribed DENV-2 replicon RNA and used to calculate the sfRNA:gRNA ratio in each sample. Ratios were plotted relative to the control knockdown and analyzed by one-way ANOVA using the GraphPad Prism software package. (**E**-**H**) Analysis of ISG induction following DENV-2 or YFV17D infection. Total cellular RNA from knockdown and infection with DENV-2 or YFV17D was analyzed by qPCR for IFITM2 or IFNB mRNA

levels normalized to GAPDH mRNA. IFITM2 (**E** and **G**) and IFNB (**F** and **H**) mRNA levels relative to the control knockdown was calculated and analyzed by one-way ANOVA using the GraphPad Prism software package.

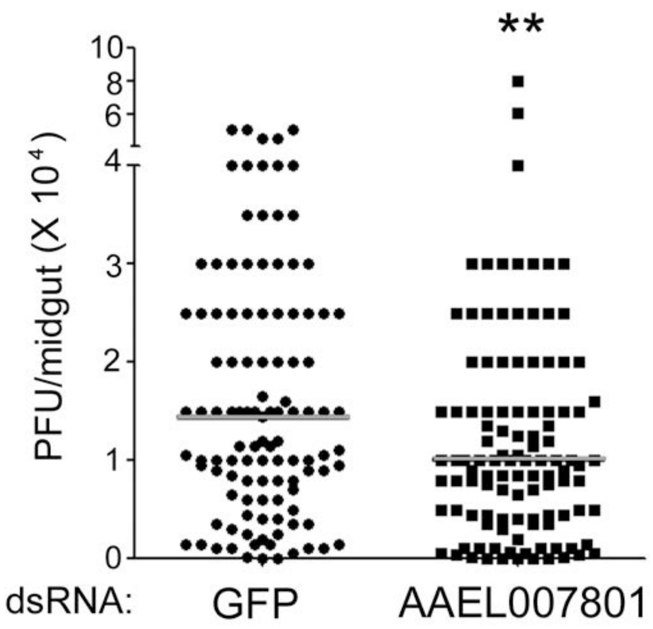

Supplementary Figure S3: Silencing of exonuclease in mosquitoes. Four day old female *Aedes aegypti* were injected with dsRNAs targeting exonuclease (AAEL007801) or GFP (control) and were then infected with DENV-2 as described in the Materials and Methods. Virus titers at 7 days post blood meal in dissected midguts are shown. Each data point represents an individual midgut. The results of four independent experiments are shown. We tested the knockdown efficiency and found that AAEL007801 RNA was reduced to 49% of the GFP control level. The Mann-Whitney U Test was applied and asterisks indicate $**p<0.01$. Grey bars indicate median values.

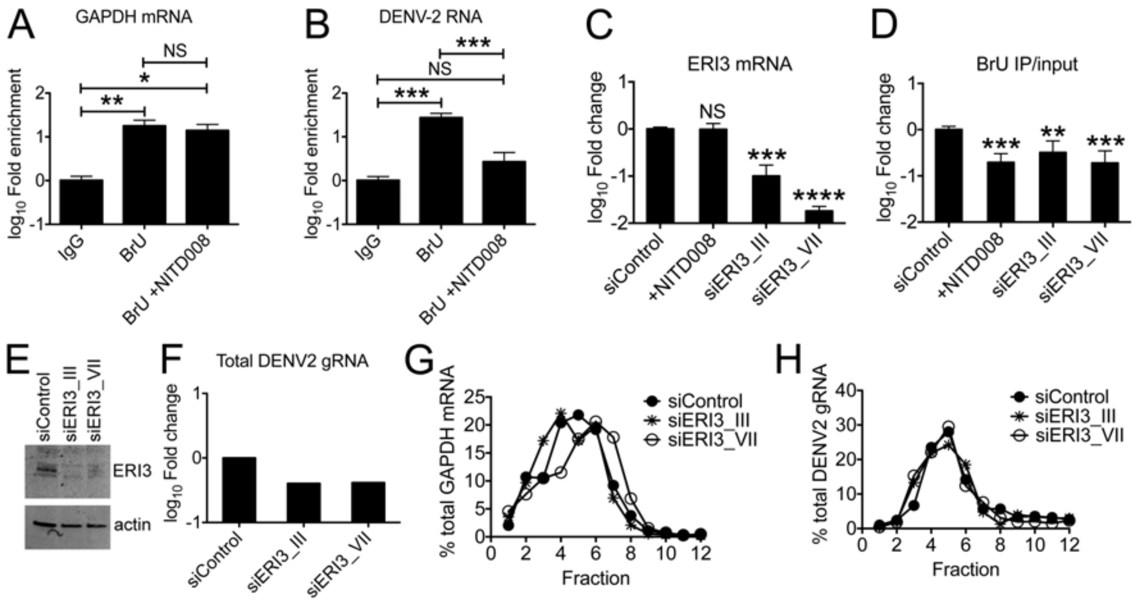

Supplementary Figure S4: Effects of ERI3 knockdown on DENV-2 RNA. (**A** and **B**) Analysis of BrU incorporation following NITD008 treatment. Cells were treated with NITD008 for 4 hours and pulse-labeled with BrU before harvesting and immunoprecipitation using a BrU-specific antibody. GAPDH (**A**) and DENV-2 (**B**) RNA levels in the pellet fractions from BrU immunoprecipitation were analyzed by qPCR and fold enrichment was calculated relative to input. Fold enrichment was plotted and analyzed by one-way ANOVA using the GraphPad Prism software package. (**C**) ERI3 mRNA levels following siRNA knockdown. HuH-7 cells were transfected with control or ERI3-specific siRNAs and incubated for 48 hours and infected with DENV-2 at an MOI of 1.0 for 16 hours. Cells were treated with NITD008 for 4 hours and pulse-labeled with BrU before harvesting and immunoprecipitation using a BrU-specific antibody. Total cellular RNA was analyzed by qPCR for ERI3 and GAPDH mRNA levels. GAPDH-normalized ERI3 mRNA levels were plotted relative to the control knockdown and analyzed by one-way ANOVA using the GraphPad Prism software package. (**D**) Analysis of

DENV-2 RNA synthesis following ERI3 knockdown. DENV-2 and GAPDH RNA from the total cell and pellet from BrU immunoprecipitation were analyzed by qPCR. GAPDH-normalized DENV-2 RNA levels from input and pellet were used to generate a ratio of IP/total RNA, plotted relative to the control knockdown and analyzed by one-way ANOVA using the GraphPad Prism software package. (**E**-**H**) Polyribosome analysis of DENV-2 RNA following ERI3 knockdown. (**E**) HuH-7 cells were transfected with control or ERI3-specific siRNAs and incubated for 48 hours prior to infection with DENV-2. 24 hours later, total cell lysates were harvested and analyzed by western blot for ERI3 and pan-actin. (**F**) Total RNA was extracted from cells and analyzed by qPCR for GAPDH and DENV-2 RNA levels. GAPDH-normalized DENV-2 RNA levels were plotted relative to the control knockdown sample. (**G** and **H**) Cytoplasmic samples were fractionated over a 10-50% continuous sucrose gradient, extracted for RNA and analyzed for GAPDH (**G**) and DENV-2 (**H**) RNA levels by qPCR. The percent of total RNA in each fraction was calculated and plotted against the fraction number for the control and ERI3 knockdown samples.

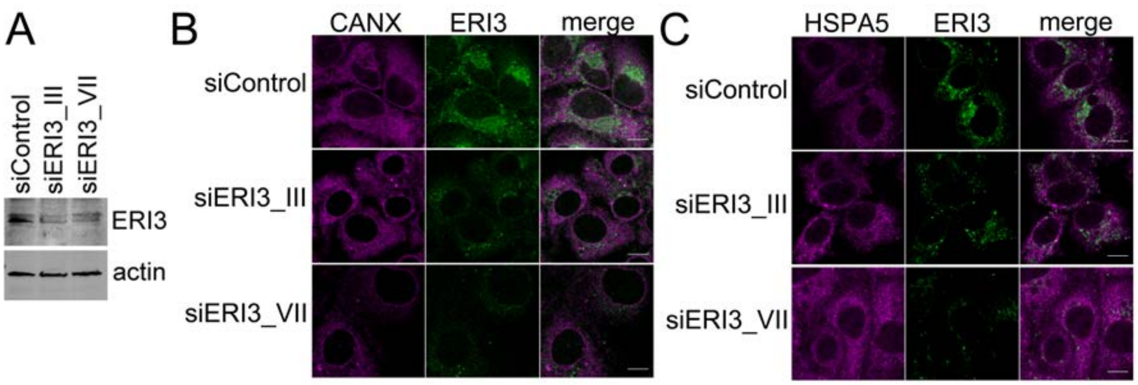

Supplementary Figure S5: Validation of ERI3 antibody for immunofluorescence microscopy studies. (**A**) Total cell lysates from HuH-7 cells transfected with control or ERI3-specific siRNAs were analyzed by western blot for ERI3 and pan-actin. (**B** and **C**) HuH-7 transfected with control or ERI3-specific siRNAs were plated on coverslips, fixed, permeabilized and probed for ERI3 and the ER markers CANX (**B**) or HSPA5 (**C**). Cells were imaged by confocal microscopy.

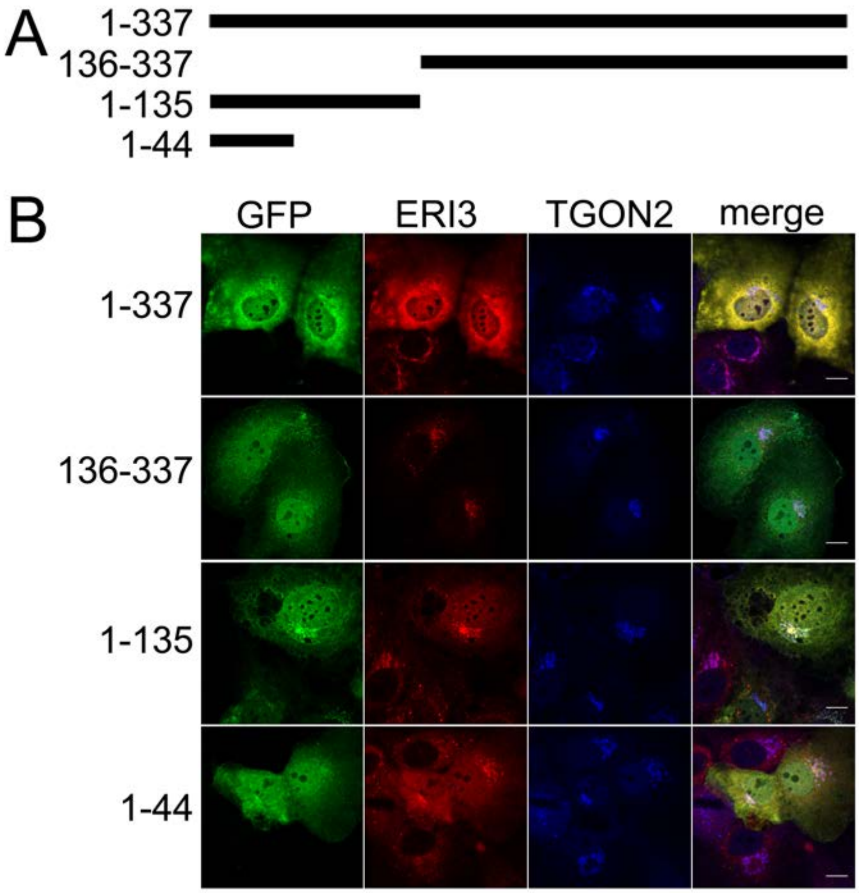

Supplementary Figure S6: Sequences in the N-terminus of ERI3 drive localization to the Golgi. (**A**) Diagram of sequences used to generated EGFP fusion constructs. The amino acid coordinates from ERI3 are indicated on the left. (**B**) HuH-7 cells were plated on coverslips and transfected with EGFP-tagged ERI3 expression constructs. The amino acid coordinates for each construct are indicated on the lefthand side of the panel. 24 hours later, coverslips were fixed, permeabilized and probed with antibodies to ERI3, EGFP and TGON2. Cells were imaged using confocal microscopy.

References

1 Yin, Z. *et al.* An adenosine nucleoside inhibitor of dengue virus. *Proceedings of the National Academy of Sciences of the United States of America* **106**, 20435-20439, doi:10.1073/pnas.0907010106 (2009).

2 Paulsen, M. T. *et al.* Use of Bru-Seq and BruChase-Seq for genome-wide assessment of the synthesis and stability of RNA. *Methods* **67**, 45-54, doi:10.1016/j.ymeth.2013.08.015 (2014).
